# Delay-induced patterns in a two-dimensional lattice of coupled oscillators

**DOI:** 10.1038/srep08522

**Published:** 2015-02-17

**Authors:** Markus Kantner, Eckehard Schöll, Serhiy Yanchuk

**Affiliations:** 1 Weierstrass Institute for Applied Analysis and Stochastics, Mohrenstr. 39, 10117 Berlin, Germany; 2 Technical University of Berlin, Institute of Theoretical Physics, Hardenbergstr. 36, 10623 Berlin, Germany

## Abstract

We show how a variety of stable spatio-temporal periodic patterns can be created in 2D-lattices of coupled oscillators with non-homogeneous coupling delays. The results are illustrated using the FitzHugh-Nagumo coupled neurons as well as coupled limit cycle (Stuart-Landau) oscillators. A “hybrid dispersion relation” is introduced, which describes the stability of the patterns in spatially extended systems with large time-delay.

Coupled dynamical systems with time-delays arise in various applications including semiconductor lasers^1^,^2^,^3^,^4^, electronic circuits^5^, optoelectronic oscillators^6^, neuronal networks^7^,^8^,^9^, gene regulation networks^10^, socioeconomic systems^11^,^12^ and many others^13^,^14^,^15^,^16^,^17^,^18^. Understanding the dynamics in such systems is a challenging task. Even a single oscillator with time-delayed feedback exhibits phenomena, which are not expected in this class of systems, such as Eckhaus instability^19^, coarsening^20^, or chimera state^21^. Some of them, like low frequency fluctuations in laser systems with optical feedback are still to be understood^22^. The situation is even more complicated when several systems are interacting with non-identical delays. In this case, somewhat more is known about some specific coupling configurations, e.g. ring^23^,^24^,^25^,^26^, and less on more complex coupling schemes^7^,^27^,^28^,^29^,^30^. Recently, it has been shown that a ring of delay-coupled systems possesses a rich variety of stable spatio-temporal patterns^23^,^24^. For the neuronal models in particular, this implies the existence of a variety of spiking patterns induced by the delayed synaptic connections.

Here we present a system with time-delayed couplings, which is capable of producing a variety of stable two-dimensional spatio-temporal patterns. More specifically, we show that a 2D regular set of dynamical systems **u***_m_*_,*n*_(*t*) (neuronal models can be used) may exhibit a stable periodic behavior (periodic spiking) such that the oscillator **u***_m_*_,*n*_(*t*) reaches its maximum (spikes) at a time *η_m_*_,*n*_, which can be practically arbitrary chosen within the period. For this, time-delays should be selected accordingly to some given simple rule. As particular cases, the synchronous, cluster, or splay states can be realized.

Our work is a generalization of the previous results on the ring^23^,^24^, extending them to the two-dimensional case. However, the analysis, which we have to employ has important differences. In particular, the combination of the spatial structure of the system (spatial coordinates *m* and *n*) and temporal delays required the introduction of a so called “hybrid dispersion relation” for the investigation of the stability of stationary state and nonlinear plane waves in the homogeneous system. Roughly speaking, this hybrid dispersion relation is a synthesis of the dispersion relation from the pattern formation theory in spatially extended systems^31^,^32^ and the pseudo-continuous spectrum developed for purely temporal delay systems^19^,^33^,^34^.

We believe that such a higher-dimensional extension allows to think about the possibility of employing such systems for generating or saving visual patterns, and can be probably of use for information processing purposes. Small arrays of delay-coupled optoelectronic oscillators have indeed already been realized experimentally^6^. Similarly, autonomous Boolean networks of electronic logic gates have been demonstrated as versatile tools for the realizations of various space-time patterns^35^. Moreover, our analysis provides another evidence that the delays in coupled systems can play a constructive functional role.

More specifically, we consider a lattice of *M* × *N* delay-coupled systems (delay differential equations) of the form
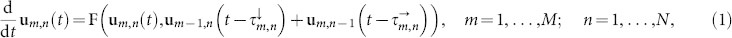
where 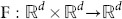 is a nonlinear function determining the dynamics of 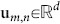 in the lattice. The indices *m* and *n* determine the position of the node, see Fig. 1. We assume periodic boundary conditions **u***_M_*_ + 1,*n*_ ≡ **u**_1,*n*_ and **u***_m_*_,*N* + 1_ ≡ **u***_m_*_,1_ such that the system has translation invariance. Time-delays 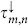 and 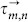 denote the connection delays between the corresponding nodes. Since each node has two incoming connections, the arrows ↓ and → correspond to the coupling from the node located above, respectively left, see Fig. 1. Here we restrict the analysis to two systems: Stuart-Landau (SL) oscillators as a simplest dynamical system exhibiting limit cycle behavior and FitzHugh-Nagumo (FHN) systems as a representative of conductance based, excitable neuronal models^36^,^37^. While the first model allows for a deeper analytical insight, the second one can be studied mainly numerically and shows qualitatively similar results.

An example of a stable spatio-temporal pattern in a lattice of 100 × 150 FHN neurons with non-homogeneous delays, the “Mona Lisa”-pattern is shown in Fig. 2. Each frame corresponds to a snapshot at a fixed time *t* and the different level of gray at a point (*m*,*n*) corresponds to the value of the voltage component of **u***_m_*_,*n*_(*t*) at this time *t*. More details on how such patterns can be created are given in the following sections.

The structure of the remaining part of the paper is as follows: In section Results we firstly consider SL systems with homogeneous time-delays 
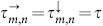
. We investigate the stability of the homogeneous steady state as well as various plane wave solutions in the system. The number of stable plane wave solutions is shown to increase with the delay. Further, similar results are obtained for the FHN systems. Afterwards, we consider the case when the delays are not identical. In this case it is shown how a variety of spatio-temporal patterns can be created by varying the coupling delays. Finally, additional illustrative examples are presented.

## Results

### Stuart-Landau oscillators with homogeneous coupling delays

In this section we start with a lattice of SL oscillators with homogeneous delays 
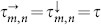
:
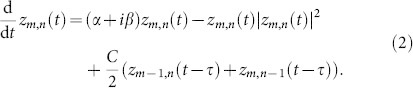
The variables *z_m_*_,*n*_ are complex-valued. The parameter *α* controls the local dynamics without coupling, i.e. a stable steady state exists for *α* < 0 and a stable limit cycle for *α* > 0; *β* is the frequency of this limit cycle. The coupling strength is determined by *C* > 0.

We firstly study the bifurcation scenario, which is associated with the destabilization of the homogeneous steady state *z* = 0 and the appearance of various plane waves. Many aspects of this scenario can be studied analytically due to the *S*^1^ equivariance of the system: **F**(*e^iν^* ·, *e^iν^* ·) = *e^iν^***F**(·, ·) for any real *ν*. At some places we will assume additionally that the delay *τ* is large comparing to the timescale of the system (we will mention it each time explicitly), which simplifies analytical calculations.

#### Stability and bifurcations of homogeneous stationary state

System (2) has the homogeneous steady state *z_m_*_,*n*_ ≡ 0. Its stability is described by the eigenvalues (see Methods for the derivation)
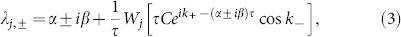
where *W_j_* is the *j*th branch of the Lambert function, 
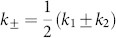
, and (*k*_1_, *k*_2_) = 2*π*(*l*/*M*, *j*/*N*), *l* = 1, …, *M*, *j* = 1, …, *N* is the wavevector. If all eigenvalues *λ_j_*_,±_ have negative real parts for all possible wavevectors (*k*_+_, *k*_−_), then the steady state is asymptotically stable.

In the case when the coupling delay is large, the discrete set of eigenvalues can be approximated by the continuous spectrum of the form (see Methods)
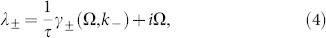
where Ω is a continuous parameter and
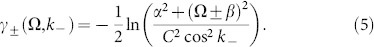


An illustration of the numerically computed eigenvalues is shown in Fig. 3 for the system of 3 × 3 coupled SL oscillators for three cases: stable, critical, and unstable. All eigenvalues accumulate along the curves *γ*_±_(Ω, *k*_−_) given by Eq. (4) with maxima at Ω = ± *β*. For a 3 × 3 lattice, only 3 values of |*k*_−_| are realized: 0, 2*π*/3, and 4*π*/3 (where the latter two are mapped on each other in the spectrum due to the cos^2^ (*k*_−_)). One can observe also how multiple Hopf-bifurcations may emerge after the destabilization. In the following section we discuss the plane waves arising in these Hopf-bifurcations.

#### Nonlinear plane waves

Because of the phase-shift symmetry of the Stuart-Landau system (2), periodic solutions emerging from the homogeneous steady state via Hopf-bifurcations have the following form
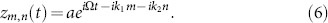
By substituting (6) into (2), we obtain the equation for amplitude *a*, frequency Ω, and the wavevector **k** = (*k*_1_, *k*_2_) of the periodic solutions
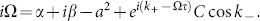
Taking real and imaginary parts, we obtain
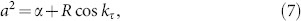

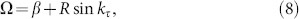
where we denote *R*: = *C* cos *k*_−_ and *k_τ_*: = *k*_+_ − Ω*τ*. By excluding *k_τ_* we obtain
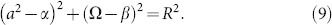
Therefore all periodic solutions can be found on circles (9) in the (*a*^2^−*α*, Ω)-parameter space. Equation (8) is known as Kepler's equation an can be solved numerically with respect to Ω. The number of solutions of (8) matches the number of Hopf-bifurcations and periodic solutions. All possible frequencies are confined to the interval −|*R*| + *β* ≤ Ω ≤ |*R*| + *β*. By studying Eqs. (7) and (8), the number of Hopf-bifurcations, or periodic solutions respectively, can be estimated as 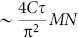 asymptotically for large *M* and *N* (we omit here the straightforward calculations). Thus, in the case of large delay or lattice-size the number of solutions grows and any point on the circles (9) refers to a periodic solution, i.e. the circle disc is densely filled with points (*a*^2^ − *α*, Ω) corresponding to the existing periodic solutions. As an example, the positions of periodic solutions in a 10 × 10-lattice are shown in Fig. 4.

The stability of plane wave solutions is studied in detail in Methods. The bifurcation diagram in Fig. 5 summarizes and illustrates the obtained results, showing the regions where the plane waves are stable (light gray), weakly unstable (darker gray, labeled with *U* and *M*), and strongly unstable (dark gray, labeled with *S*).

The main qualitative conclusions of the plane waves analysis are as follows: The family of plane wave solutions (6) is located on the circles (9) (for a fixed *k*_−_ or *R*, respectively), and the number of plane waves grows as the product ~ *τMN*. The stability of a plane wave is governed by the characteristic equation (23) and determined by its position on the circle. More specifically, the plane waves with the higher amplitude tend to be more stable than those with the lower amplitude. Figure 4 and 5 illustrate this by showing stable, as well as weakly and strongly unstable “positions” on the circle. Thus, with increasing *α*, the number of stable plane waves increases. Plane waves with smaller |*k*_−_| also tend to be more stable than those with larger |*k*_−_|. Therefore we expect that the plane waves which are almost diagonal are more abundantly observed.

### FitzHugh-Nagumo neurons with homogeneous coupling delays

**Figure 1:**
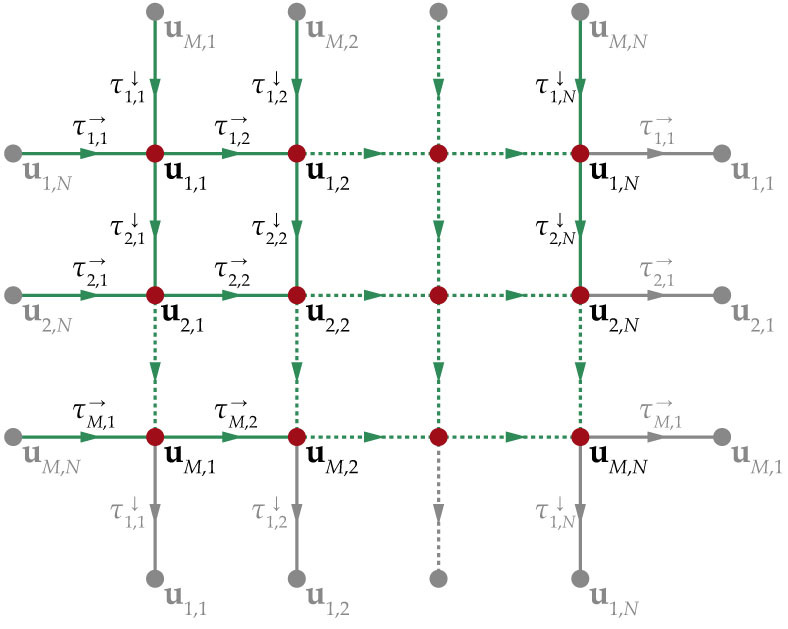
Coupling scheme. We consider a two-dimensional lattice of delay-coupled oscillators with translation-invariance in both spatial directions (a discrete 2-torus). The dynamics of each node **u***_m_*_,*n*_(*t*) is described by system (1). Each coupling connection possesses a delay 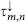 or 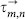. All edges are unidirectional.

In this section we consider a lattice of *M* × *N* delay-coupled FitzHugh-Nagumo neurons, which are coupled via excitatory chemical synapses. The coupling architecture is the same as described in Fig. 1. The model system reads
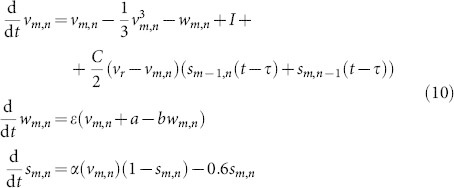
with 
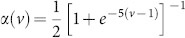
. The variable *v_m_*_,*n*_ denotes the membrane potential of the corresponding neuron and *w_m_*_,*n*_ is a slow recovery variable, combining several microscopic dynamical variables of the biological neuron. The external stimulus current applied to the neuron is denoted by *I* and *C* is the coupling strength. We fix the parameters *a* = 0.7, *b* = 0.8, and *ε* = 0.08. The reversal potential is taken as *v_r_* = 2 for excitatory coupling. Similar model equations have been investigated in Refs. 24, 38 for unidirectional rings.

We demonstrate that the destabilization of the homogeneous steady state, the set of plane waves as well as their stability properties possess the same qualitative features which we observed in the Stuart-Landau system (2). However, the apparent difficulty for the analysis of nonlinear plane waves is that they are not known analytically. Therefore we use numerical bifurcation analysis with DDE-Biftool^39^ and have to restrict ourselves to relatively small lattice size and delay values.

#### Homogeneous steady state and its stability

The system (10) has a homogeneous steady state 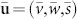. The value for the membrane resting potential 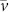 can be obtained as a solution of the scalar equation
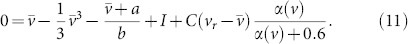
The steady-state values of the remaining variables follow as 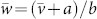 and 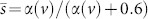. In the case of weak coupling strength the homogeneous stationary state is unique, but for *C*_SN_ = 1.46475 a saddle-node bifurcation of the equilibrium takes place. For strong coupling *C* > *C*_SN_ there is a domain of the control parameter *I* with three coexisting stationary states, see Fig. 6.

In Methods, the characteristic equation, which determines the stability of the homogeneous state, is derived (Eq. (32)) and studied. The resulting bifurcation diagram is shown in Fig. 6 together with the asymptotic spectra in the case of large delay and lattice size. The boundaries of domains, where Hopf-bifurcations are possible are shown as *H*_1_ and *H*_2_.

#### Hopf-bifurcations and periodic attractors

Using the software package DDE-Biftool^39^, we perform a continuation of the Hopf-bifurcations in the (*I*, *τ*)-plane. The result is shown in Fig. 7, where the Hopf-frequency Ω is plotted vs. the time-delay *τ*. The structure of the branches can be understood by using reappearance arguments for periodic solutions^40^. Some of the Hopf-branches terminate with zero frequency in a homoclinic bifurcation.

We perform also a numerical continuation of the periodic solutions, emerging from the Hopf-bifurcations. The spatial orientation of a periodic solution is conserved along the branch, while varying the external current *I* as a control parameter. Typically, a periodic solution connects two Hopf-points, which are both solutions of Eq. (32) with the same *k*_+_ and *k*_−_. For vanishing delay, all stable periodic orbits are diagonal traveling waves with *k*_−_ = 0, including the synchronized solution. Increasing the coupling delay significantly enhances the stability properties of periodic solutions and allows for stable traveling waves with *k*_−_ ≠ 0. Moreover, the periodic solutions appear in a larger regime of the control parameter *I*. Snapshots of several coexisting traveling waves in a system of 100 × 100 FHN-neurons with *τ* = 50 are shown in Fig. 8. Such solutions serve as the starting point for the more complicated patterns in systems with inhomogeneous delays, discussed in the following section.

### Patterns in systems with inhomogeneous delays

#### Componentwise time-shift transformation

Consider a delayed dynamical system with a coupling topology as described by Eq. (1) with homogeneous delays. In the previous section we have shown the existence and stability properties of traveling wave patterns in the Stuart-Landau system, which have the explicit form given by Eq. (6). In the case of FitzHugh-Nagumo oscillators the existence of patterns of the form **u***_m_*_,*n*_(*t*) = **v**(*t* − *T*(*k*_1_*m* + *k*_2_*n*)/2*π*) was demonstrated numerically. In both systems, there is a large number of stable coexisting periodic patterns that grows with the increasing time-delay and the number of oscillators in the lattice.

Here we show how one can transform the plane waves of the homogeneous system into an (almost) arbitrary pattern by adjusting the coupling delays. The derivation of the transformation presented here is a generalization of the method described in Refs. 23, 24 for unidirectionally coupled rings and Ref. 27 for arbitrary networks with delays.

Rewriting system (1) with respect to the new coordinates **v** given by **u***_m_*_,*n*_(*t*) = **v***_m_*_,*n*_(*t* − *η_m_*_,*n*_) leads to the new system
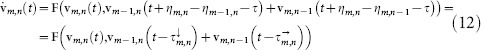
(see Eq. (1)) with the adjusted non-homogeneous delays
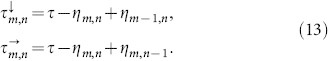
The time-shifts 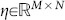 can have an arbitrary form, up to the restriction that the new delays need to be positive. It is important to note, that the round-trip time in each direction is conserved by this transformation. By adjusting the time-shifts, one can obtain in system (12) stable, time-periodic attractors of various spatial forms. For example, a stable synchronous periodic solution **u***_m_*_,*n*_(*t*) = **u**_0_(*t*) = **u**_0_(*t* + *T*) of the homogeneous system corresponds to a solution **v***_m_*_,*n*_(*t*) = **u**_0_(*t* + *η_m_*_,*n*_) in the non-homogeneous system, where each component is shifted in time by *η_m_*_,*n*_. E.g. in the case of Stuart-Landau oscillators, the transformation *z_m_*_,*n*_(*t*) = *Z_m_*_,*n*_(*t* − *η_m_*_,*n*_) yields the explicit form
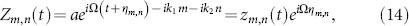
with *z_m_*_,*n*_(*t*) from Eq. (6) solving the problem with homogeneous delays (2). The stability properties of the periodic solutions are invariant with respect to the componentwise time-shift transformation, i.e. the characteristic exponents do not change. We refer to Ref. 27 for a more detailed analysis of the stability.

The time-shift will result to a shifted value of the dynamical variables (e.g. voltage for the neuronal models). Thus, the encoded pattern *η_m_*_,*n*_ will be visible in the dynamical variables of the ensemble. Since *η_m_*_,*n*_ is practically arbitrary, there is a variety of patterns, which can appear as stable attractors in the systems with inhomogeneously delayed connections. Here and in the examples given later, we focus on patterns that arise from the spatially homogeneous solution **u***_m_*_,*n*_(*t*) = **u**_0_(*t*). This is done for the sake of simplicity and because of the favorable stability properties of the synchronous solution (it has the spatial mode *k*_−_ = 0). Note that, since the number of patterns is not affected by the transformation, there can be coexisting stable transformed traveling wave patterns 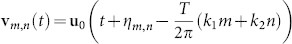
for admissible values of the wavevector (*k*_1_, *k*_2_). In order to obtain a particular pattern in a numerical simulation, one has to properly adjust the initial functions according to the desired pattern. As a rule, the delay-times should be kept as small as possible (however, still having the new delays (13) positive) to limit the number of coexisting stable patterns and therefore enhance the convergence.

### Examples of created patterns

**Figure 2:**
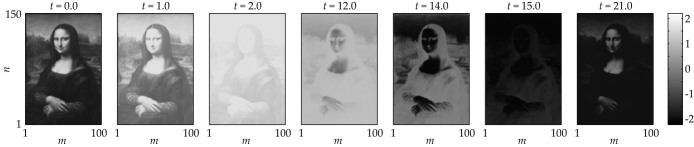
Example of a created spatio-temporal pattern. Snapshots of the spatio-temporal behavior in a system of 100 × 150 identical FHN neurons Eq. (10) with appropriately adjusted time-delays 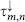 and 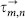. At each grid point with the coordinate (*m*, *n*), the level of gray (see colorbar) corresponds to the membrane voltage *v_m_*_,*n*_(*t*) at this time moment. The pattern reappears periodically with a time period *T* = 21.95. More details are given in Results.

**Figure 3:**
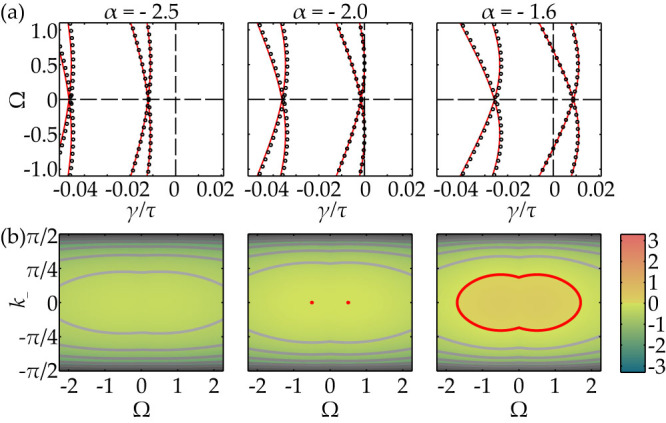
(a) Eigenvalues of the homogeneous steady state for SL system. For 3 × 3 lattice of delay-coupled SL oscillators with *C* = 2, *β* = 0.5, and *τ* = 20, the plots in (a) show numerically computed eigenvalues (3) and the continuous large delay approximation (4) by the red line. The stationary state is stable for *α* = −2.5, critical at *α* = −2, and unstable for *α* = −1.6. (b) Color plots of *γ*(Ω, *k*_−_) ≈ Re [*λτ*] as a function of Ω and *k*_−_. The parameter *α* is the same as in (a). For large lattices, all values of *k*_−_ can be realized.

**Figure 4:**
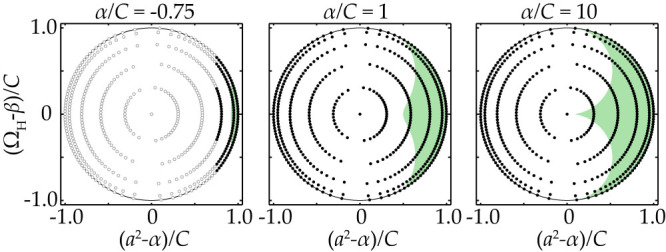
Hopf-bifurcation points and periodic solutions. The black dots show the positions of all periodic solutions (or Hopf-bifurcation points respectively) of the SL-system (2) in the parameter-plane of a 10 × 10 lattice with *β* = 0.5, *C* = 2 and *τ* = 10 for different values of *α*. The empty gray dots represent unborn periodic solutions (*α* too small). For large *M*, *N* and *τ* the disc becomes densely filled with periodic solutions. The green area marks the stable regions on the disc for the respective value of *α* according to Eq. (30). The stable domain grows with increasing *α*. In the limit of infinite *α* one quarter of all existing periodic solutions are stable.

Illustrative examples of stable spatio-temporal patterns in a lattice with non-homogeneous delays are shown in Figs. 2 and 9. All examples are constructed from synchronized solutions with **k** = (0, 0)*^T^* via the delay-transformation (13). However, the scaling of the patterns *η_m_*_,*n*_ with respect to the period time is different in the examples. In the “Mona Lisa”-pattern (Fig. 2) the spiking times are chosen only slightly different, so that the pattern is a slightly adapted standing front solution. In the examples in Fig. 9 the spiking-times are distributed over the whole period.

## Discussion

We have shown that arbitrary stable spatio-temporal periodic patterns can be created in two-dimensional lattices of coupled oscillators with inhomogeneous coupling delays. We propose that this offers interesting applications for the generation, storage, and information processing of visual patterns, for instance in networks of optoelectronic^6^ or electronic^35^ oscillators. Our results have been illustrated with two models of the local node dynamics which have a wide range of applicability: (i) the Stuart-Landau oscillator, i.e., a generic model which arises by center-manifold expansion of a limit cycle system near a supercritical Hopf-bifurcation, and (ii) the FitzHugh-Nagumo model, which is a generic model of neuronal spiking dynamics.

## Methods

### Characteristic equation for the homogeneous state in the coupled SL systems

System (2) has a homogeneous steady state *z_m_*_,*n*_ ≡ 0. We investigate the stability of this stationary state and find the expression (3) for the eigenvalues as well as the large delay approximation (5). Linearizing the equation of motion (2) at *z_m_*_,*n*_ = 0 yields the following equation for the evolution of small perturbations *δz_m_*_,*n*_(*t*):
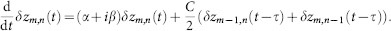
This equation can be diagonalized by a spatial discrete Fourier-transformation
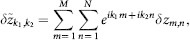
where the wavevector **k** = (*k*_1_, *k*_2_) admits the following discrete values:
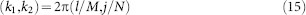
with *l* = 1, …, *M* and *j* = 1, …, *N*. We obtain
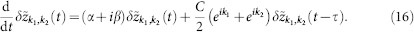
Since the equation for the Fourier modes is uncoupled, one can drop the indices *k*_1_ and *k*_2_ for simplicity (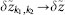) and introduce the notations
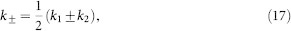
which is basically a rotation of coordinates in the Fourier space. Note that *k*_±_ admits discrete values accordingly to (15). Then system (16) can be rewritten in real coordinates 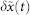, 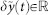 as
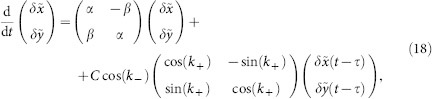
where we decomposed the complex variable as 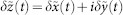. Since the modes are decoupled in the Fourier space, the corresponding characteristic equation factorizes and reads
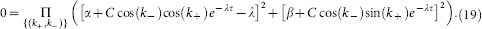
The solution of this transcendental equation with respect to *λ* leads to the expression (3).

#### Large delay approximation

A deeper analysis of the spectrum can be achieved for large delays using the asymptotic methods described in Refs. 17, 33, 41. Accordingly to these results, the spectrum splits generically into two parts for large delays. The first part is called the *strongly unstable spectrum* and the second part is the *pseudo-continuous spectrum*. The strong spectrum exists for *α* > 0 and consists of two complex conjugate, isolated roots which are close to *λ_S_*_,±_ = *α* ± *iβ*. In such a case, the contribution of the term 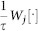 in (3) vanishes. In fact, the strong spectrum always converges to the unstable part of the spectrum of the system with omitted delayed terms^33^,^34^,^42^, i.e. 
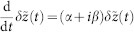
 in this case. Besides the strong spectrum there are infinitely many more eigenvalues, accumulating on some curves in the complex plane as *τ* → ∞. These eigenvalues form the pseudo-continuous spectrum and can be found by substituting the ansatz 
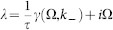
 in the characteristic equation (19). One obtains the two branches
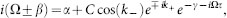
where the small term *γ*/*τ* has been neglected. It can be solved as
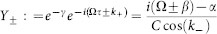
and finally we obtain 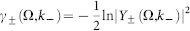, which leads to Eq. (5). Note that the spectrum is invariant with respect to complex conjugation, i.e. *γ*_+_ (−Ω, *k*_-_) = *γ*_−_ (Ω, *k*_−_). It is easy to see that the spatial mode *k*_−_ = 0 corresponds to the maximal values of *γ*(Ω, *k*_−_). Thus, the spatial modes with *k*_−_ = 0 are most unstable. Moreover, it is easy to check that the maxima of *γ*_±_ are negative for |*α*| > *C* and positive otherwise. This implies that the homogeneous steady state is asymptotically stable for *α* < −*C* and unstable for *α* > −*C* (we take also into account that there is a strongly unstable spectrum for *α* > 0). Hence, the destabilization takes place at *α* = −*C* via Hopf-bifurcation at the frequencies Ω ≈ ±*β* for a perturbation with *k*_−_ = 0.

### Stability of plane wave solutions in coupled SL system

The local asymptotic stability of plane wave solutions can be studied using the linearized equation for small perturbations *ξ_m_*_,*n*_(*t*). In co-rotating coordinates we set
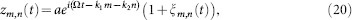
where the plane wave is recovered by the steady state *ξ_m_*_,*n*_ = 0. Therefore, after substituting (20) in (2) and linearizing the obtained equation in small perturbations *ξ_m_*_,*n*_(*t*), we obtain
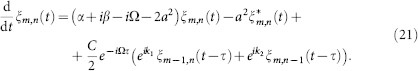
The solutions can be found by the ansatz
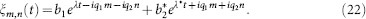
The ansatz (22) can be obtained, e.g. by rewriting the system (21) in the real form, diagonalizing it with the discrete Fourier transform, and noticing that the equations for the Fourier components 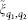 and 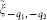 are complex conjugate, and hence, they have the same stability properties with the complex conjugate eigenvalues, see also Ref. 32. After substituting (22) into (21), the coefficients at the two linearly independent functions 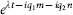 and 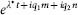 should be zero. This leads to the system of two linear equations for unknowns *b*_1_ and *b*_2_, which we omit here for brevity. This system has a nontrivial solution if the determinant of its matrix is zero. As a result, we arrive at the following characteristic equation
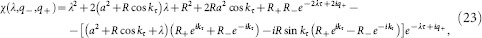
where we introduced 
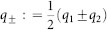
 and *R*_±_: = *C* cos(*k*_−_ ± *q*_−_). The obtained characteristic equation (23) determines the stability of a plane wave. Namely, for any plane wave, which is defined by the amplitude *a*, frequency Ω, wavevectors *k*_+_ and *k*_−_ (then also *k_τ_* = *k*_+_ − Ω*τ* is given), the equation (23) determines the stability with respect to the perturbation mode with the spatial perturbations *q*_+_ and *q*_−_. In particular, if for all *q*_+_, *q*_−_ ∈ [0, 2*π*], all the solutions *λ* of the characteristic equation (23) have negative real parts, then the plane wave is asymptotically stable. The symmetry-relation χ*(λ*, *q*_−_, −*q*_+_; −*k_τ_*) = *χ*(*λ*, *q*_−_, *q*_+_; *k_τ_*) implies, that the stability properties of periodic solutions are invariant with respect to changing *k_τ_* → −*k_τ_*. Notice that the obtained equation is a quasi-polynomial, which has generically infinitely many roots.

Although Eq. (23) can be studied numerically for each given set of parameters, an additional useful analytical insight in the properties of its solutions is possible under the assumption of large delay *τ*. This is performed in the next section.

### Plane wave solutions in coupled SL system: large delay approximation

#### Strong spectrum

**Figure 5:**
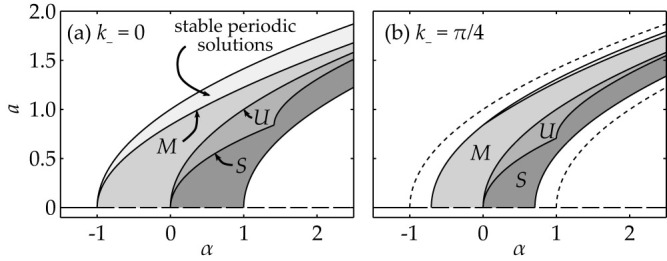
Stability diagrams for plane waves of the SL-system (2) with different values of *k*_−_: (a) *k*_−_ = 0 and (b) *k*_−_ = *π*/4. Stable plane waves are shown by a light gray and are located above the *M*-labeled curve in this projection. The curves denote the boundaries of the different stability regimes discussed in the text: *M* modulational instability, *U* uniform instability, and *S* strong instability. The size of the stable regime decreases with the increasing of *k*_−_.

As it was discussed previously, the strong spectrum involves only the instantaneous part of the dynamics. Therefore it does neither depend on the spatial perturbation modes **q** nor on the network-size, since all spatial effects induced by the coupling-structure are contained in the delayed terms. The reduced characteristic equation for the strong spectrum can be formally obtained by setting 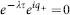 in (23):
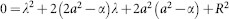
Its solutions are
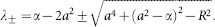
Any of the solutions *λ*_±_ with positive real part belongs to the strong spectrum. Simple calculations show that there exists at least one strong eigenvalue with positive real part if
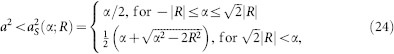
i.e. if the amplitude of the plane wave is smaller than the one determined by the curve *a_S_*(*α*, *R*). Moreover, when the inequality *a*^4^ + (*a*^2^ − *α*)^2^ − *R*^2^ < 0 is satisfied, there are two complex conjugate unstable eigenvalues 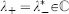 with Re *λ*_±_ = *α* − 2*a*^2^. The bifurcation diagram in Fig. 5 illustrates the regions of strong instability of plane waves (dark gray regions, labeled with *S*).

#### Pseudo-continuous (weak) spectrum

Besides the strong spectrum, there are infinitely many eigenvalues in the *weak* or *pseudo-continuous spectrum*. Similarly to the case of the steady state, this spectrum can be found by substituting the ansatz
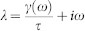
into the characteristic equation (23). In the limit of large delay, the terms of the order 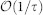 can be neglected, resulting in the following equation
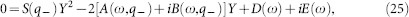
with 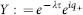, and the real valued functions
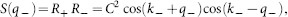

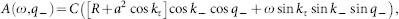

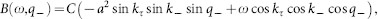

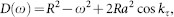

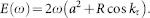
Solving the quadratic equation (25) with respect to *Y* leads to
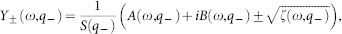
with
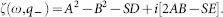
Note that the solutions do not depend on *q*_+_, which therefore has no impact on the stability in the limit of large delay. Since there are two solutions *Y*_±_, one obtains two branches of the pseudo-continuous spectrum
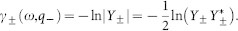
The spectrum possesses the following symmetries
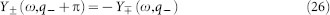
and
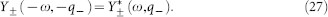
The first relation (26) implies that it is sufficient to consider only one of the two functions *γ*_±_(*ω*, *q*_−_), since they are related to each other by the shift *q*_−_ → *q*_−_ + *π* as
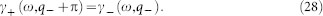
This also indicates that the pseudo-continuous spectrum is twofold degenerate in the limit of *M*, *N* → ∞. The second property (27) implies that the spectrum has the reflection-symmetry in the (*ω*, *q*_−_)-plane:
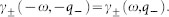
Note that in the special case *k*_−_ = 0 the additional symmetry-relations 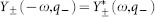 and *Y*_±_(*ω*, −*q*_−_) = *Y*_±_(*ω*, *q*_−_) hold.

The eigenvalues *λ* are known as *characteristic exponents* or *Floquet-exponents* and are related to the *Floquet-multipliers* via 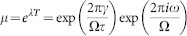. As known from the Floquet-theory for periodic solutions, there is always one trivial multiplier *μ* = 1 or trivial exponent *λ* = 0, arising from the continuous symmetry with respect to time-shifts in autonomous systems (phase shift on the limit cycle). For a perturbation with *ω* = 0 and *q*_−_ = 0 one obtains
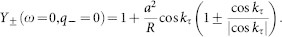
The corresponding trivial characteristic exponent follows as
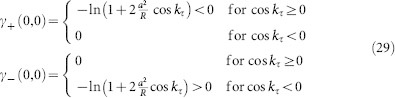
Note that this property of the spectrum is not affected by the long delay approximation, since the approximation becomes exact at *γ* = 0. Apparently there are two parameter domains separated by cos *k_τ_* = 0. Using (7), one finds that this boundary corresponds to the the curve 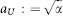 (see *U* in Fig. 5). Thus, all periodic solutions with the amplitudes smaller than *a_U_* for a given *α* are unstable due to a positive characteristic exponent with *ω* = 0. According to Ref. 31 this instability is called a *uniform instability*. In order to determine the neutral stability curve, the following discussion is restricted to the regime with cos *k_τ_* ≥ 0. Since the relation (28) holds, we will focus the analysis on *γ*_−_(*ω*, *q*_−_).

The trivial multiplier always denotes a critical point of the pseudo-continuous spectrum at (*ω* = 0, *q*_−_ = 0), where the gradient vanishes:
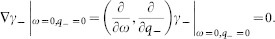
This can be verified by a direct calculation. Therefore the point (*ω* = 0, *q*_−_ = 0) is either an extremum or saddle of the pseudo-continuous spectrum. Analyzing the shape of the spectrum close to the trivial multiplier shows the appearance of the *modulational instability*^31^,^32^. For this, let us consider the second order approximation of *γ*_−_ at (*ω*, *q*_−_) = 0, involving the corresponding Hessian matrix *H*. Direct calculation leads to the following expressions for the elements of the Hessian matrix
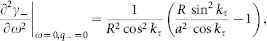

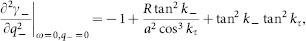

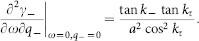
The curvature of the asymptotic continuous spectrum close to the trivial multiplier is directly related to the stability of the corresponding plane wave. If the surface is locally concave close to (*ω*, *q*_−_) = (0, 0), then the Hessian is negative definite and the corresponding periodic orbit is stable (at least the part of the spectrum which is close to (*ω*, *q*_−_) = (0, 0)). Otherwise, if the curvature is convex (Hessian is positive definite) or the origin is a saddle-point (Hessian is indefinite), the plane wave is unstable. The curvature is characterized by the real eigenvalues of the symmetric Hessian matrix. The analysis of the eigenvalues of the Hessian matrix leads to the following condition for the stability of the plane wave, which is the condition for the negativeness of the eigenvalues of *H*:
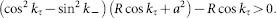
Using the amplitude relation (7), the bifurcation is described by a 3rd order polynomial in *a*^2^:
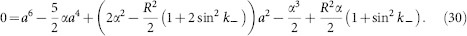
Solving Eq. (30) for *a*^2^ gives the neutral stability curve for an arbitrary plane wave with 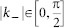 (shown as *M* in Fig. 5 for different values of *k*_−_). The analytical solution can be found by using Cardano's method, but is not written here for brevity. In the particular case *k*_−_ = 0, the neutral stability curve can be simply expressed as
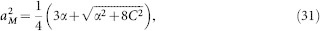
which coincides with the result obtained in Ref. 43 for the ring of coupled oscillators. For large delay a plane wave is asymptotically stable, if its amplitude exceeds the critical amplitude implicitly given by Eq. (30). By substituting (7) into (30), one can obtain the minimal *α* = *α*_0_ with
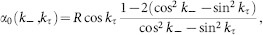
where a plane wave with particular *k*_−_ and *k_τ_* stabilizes.

Finally, we can analytically determine the position of the dominant Floquet exponent of a newly born periodic solution at its Hopf-point (*a*^2^ = 0) for *q*_−_ = ±*k*_−_ and *ω* = ±*R* sin (*k_τ_*) for *k*_−_, *k_τ_* ∈ [0, *π*/2]. This implies that the new born, unstable traveling waves tend to lose their stability in the *q*_−_ ≈ ±*k*_−_ direction.

### Stability of the homogeneous state of the coupled FHN systems

In order to analyze the stability of the stationary state, we derive the linearized evolution equation for small perturbations of the equilibrium and subsequently diagonalize it in Fourier-space, just as in the previous section. One obtains the system
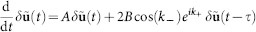
with the real valued matrices
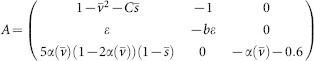
and
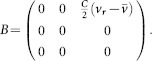
The corresponding characteristic equation reads
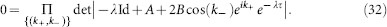
Similarly to the previous analysis, the stability of the homogeneous steady state is completely determined by Eq. (32), which can be studied numerically using e.g. Newton-Raphson iteration. An additional insight in the properties of the spectrum can be given using the large delay approximation, which is done in the following.

### Large delay approximation

#### Strongly unstable spectrum

The strongly unstable spectrum results from considering only the instantaneous part of Eq. (32)
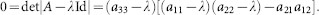
There is always one real solution *λ*_0_ = *a*_33_ ≤ −0.6 of this 3rd-order polynomial, which is strictly negative. The remaining eigenvalues are
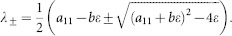
Note that all relevant parameters are contained in 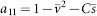, involving the current *I* and coupling strength *C* directly or via the corresponding homogeneous steady state, respectively.

**Figure 6:**
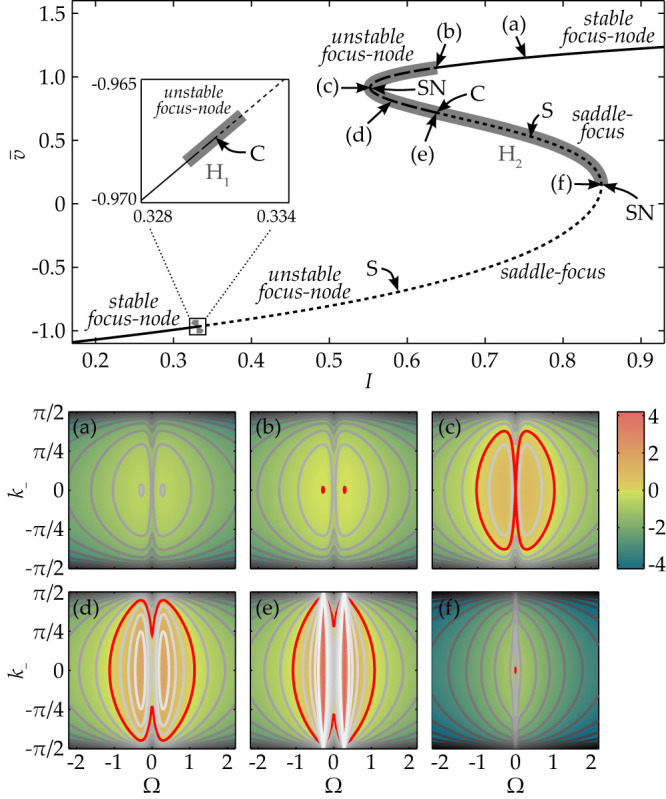
Homogeneous stationary state and its bifurcations for *C* = 3 for the FitzHugh-Nagumo model Eq.(10). Top panel: branch of the steady states solutions as a function of the current *I*. Solid line shows stable part. Bottom panel: spectrum (Eq. (33)), as a function of Ω and *k*_−_ for different points on the branch: (a) stable state, (b) destabilization via Hopf-bifurcation, (c) saddle-node bifurcation (SN), where an eigenvalue with Ω = 0 becomes unstable, (d) weakly unstable, (e) cusp bifurcation, (f) saddle-node bifurcation and the lower boundary of the Hopf-domain *H*_2_.

**Figure 7:**
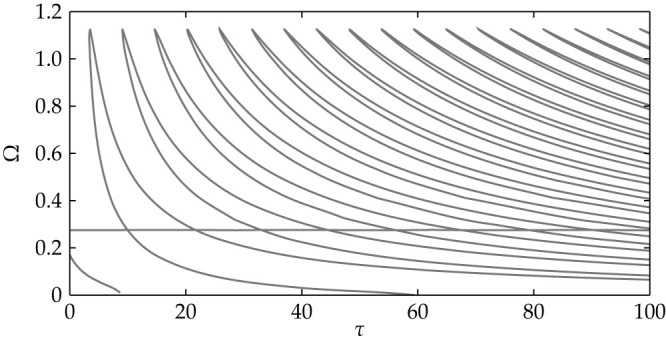
Hopf-bifurcation branches of a single FHN oscillator with delayed feedback and *C* = 3. For *M* × *N* lattices similar structures can be obtained.

**Figure 8:**
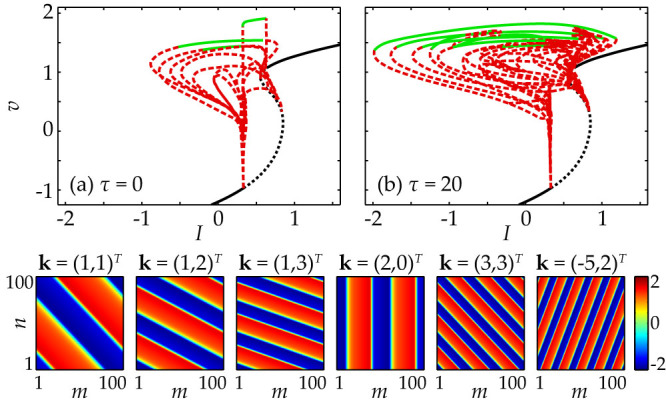
Plane waves in the homogeneous lattice of FHN oscillators. Top panel: Bifurcation diagrams for the 3 × 3-lattice of coupled FHN neurons with *C* = 3 and different delays: (a) *τ* = 0 and (b) *τ* = 20. Green solid lines denote stable periodic solutions, red dashed lines show unstable ones. Stationary state is depicted by a black line. Bottom panel: Snapshots of several coexisting stable traveling waves in a 100 × 100-lattice of delay-coupled FHN neurons with *C* = 3, *I* = 0 and *τ* = 50. The color denotes the value of the membrane voltage *v_m_*_,*n*_ of the corresponding neuron.

**Figure 9:**
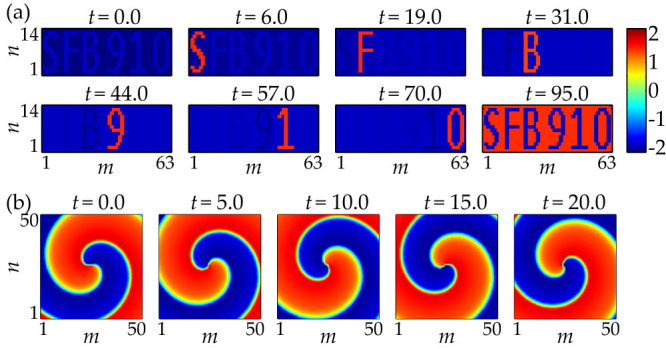
Examples of patterns created by coupling delays. Different frames show different instances of time. Top: “SFB 910”-pattern in a 63 × 14-lattice of delay-coupled FHN-neurons with *C* = 3 and *I* = −2. Bottom: spiral wave pattern in a 50 × 50-lattice of delay-coupled FHN-neurons with *C* = 3 and *I* = 0. The patterns periodically reappear with time.

The strongly unstable spectrum exists when the real part of the largest eigenvalue *λ*_+_ is positive. This is the case when *a*_11_ > *bε*. The appearance of the strongly unstable spectrum occurs at the cusp-bifurcation of the asymptotic continuous spectrum and is labeled as “C” in Fig. 6. Moreover, there exists a pair of complex conjugate eigenvalues for 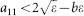, otherwise the eigenvalues are real. The corresponding boundary is labeled with “S” in Fig. 6 and mediates the transition between an unstable focus-node and a saddle-focus.

#### Pseudo-continuous spectrum

The primary bifurcations of the steady state are captured by the pseudo-continuous spectrum. Just as in the previous section in the case of Stuart-Landau oscillators, this can be found by applying the ansatz *λ* = *γ*/*τ* + *i*Ω. By neglecting terms of order 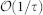 and introducing 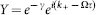, one obtains the modified characteristic equation
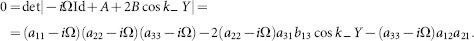
Due to the simple linear coupling-structure, this is a linear equation in *Y*, which can be solved as
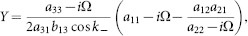
leading to the asymptotic spectrum
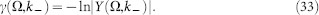
This is a function of two parameters, determining the spectrum and stability of the steady state with respect to the perturbations with the spatial mode *k*_−_ (independent of *k*_+_) and the delay-induced temporal modes Ω. Some plots of this surface are illustrated in Fig. 6. Apparently the destabilization is similar to the case of Stuart-Landau oscillators. The asymptotic weak spectrum is invariant with respect to Ω → −Ω, *k*_−_ → −*k*_−_ and *k*_−_ → *k*_−_ + *nπ*, 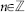. The bifurcations of (33) lead to the boundaries of domains, where Hopf-bifurcations are possible (shown as *H*_1_ and *H*_2_ in Fig. 6), and saddle-node-bifurcations. Many properties (such as extrema and roots) of the hybrid dispersion relation (33) are analytically accessible, but not given here explicitly, since they involve solutions of 3rd order polynomials.

## Author Contributions

All authors (S.Y., M.K., E.S.) wrote the main manuscript text, discussed the results, and drew conclusions. M.K. and S.Y. prepared figures. S.Y. proposed the idea and methods. M.K. performed the calculations.
